# The relationship between the body and air temperature in a terrestrial ectotherm

**DOI:** 10.1002/ece3.11019

**Published:** 2024-02-13

**Authors:** Alexandra S. Gardner, Ilya M. D. Maclean, Rolando Rodríguez‐Muñoz, Paul E. Hopwood, Kali Mills, Ross Wotherspoon, Tom Tregenza

**Affiliations:** ^1^ Centre for Ecology and Conservation University of Exeter Penryn Cornwall UK; ^2^ Environment and Sustainability Institute University of Exeter Penryn Cornwall UK

**Keywords:** air temperature, biophysical model, body temperature, climate change, ectotherm, thermal performance

## Abstract

Ectotherms make up the majority of terrestrial biodiversity, so it is important to understand their potential responses to climate change. Often, models aiming to achieve this understanding correlate species distributions with ambient air temperature. However, this assumes a constant relationship between the air temperature and body temperature, which determines an ectotherm's thermal performance. To test this assumption, we develop and validate a method for retrospective estimation of ectotherm body temperature using heat exchange equations. We apply the model to predict the body temperature of wild field crickets (*Gryllus campestris*) in Northern Spain for 1985–2019 and compare these values to air temperature. We show that while air temperature impacts ectotherm body temperature, it captures only a fraction of its thermal experience. Solar radiation can increase the body temperature by more than 20°C above air temperature with implications for physiology and behaviour. The effect of solar radiation on body temperature is particularly important given that climate change will alter cloud cover. Our study shows that the impacts of climate change on species cannot be assumed to be proportional only to changing air temperature. More reliable models of future species distributions require mechanistic links between environmental conditions and thermal ecophysiologies of species.

## Graphical abstract

The relationship between the body temperature and air temperature 1985–2019. While the air temperature impacts the ectotherm body temperature, it captures only a fraction of its thermal experience. The body temperature is not predictable from air temperature alone, and differences between the air and body temperature (temperature offset) varies between years.

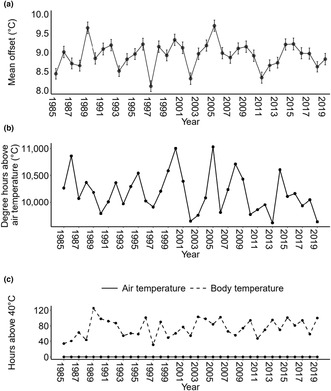

## 1 INTRODUCTION

Global air temperatures will rise over the next century due to anthropogenic climate change (IPCC, 2013). This will have profound consequences for global biodiversity because of the temperature dependence of organism physiology (Buckley et al., 2001; Somero, 2005). Rising air temperatures are particularly significant for ectotherms because they have a limited ability to regulate their own body temperature. Temperature‐dependent physiological processes will thus track environmental fluctuations unless the individual actively engages in thermoregulation (Angilletta, 2009). In the face of novel thermal conditions, they must move, adapt, or risk extinction (Nogués‐Bravo et al., 2018).

Ectotherms make up the majority of terrestrial biodiversity (Wilson, 1992), and so the impacts of climate change on ectotherms have implications at organismal, community, and ecosystem levels. To support conservation decision‐making, it is important to be able to predict how species will respond to climate change (Guisan et al., 2013). Thus far, however, this has proved difficult (e.g., Clusella‐Trullas et al., 2011; Lehmann et al., 2020). Although we know that ectotherms can respond to climate change both behaviourally and through physiological adjustments to altered environmental conditions, there is an urgent need for confidence in the accuracy of models on how this will impact distributions (Buckley et al., 2010; Sears et al., 2019).

A major cause of uncertainty as to exactly how, when, and why species respond to environmental conditions stems from the fact that most predictive models do not capture the mechanisms by which species respond to climate (Howard et al., 2023). For several decades, approaches that relate the occurrence or abundance of species with ambient air temperatures have been used to predict species' responses to altered environmental conditions (Busby, 1988; McDonald & Brown, 1992; Thomas et al., 2004). This approach tacitly assumes that body temperature, which determines the thermal performance of organisms, is directly related to ambient air temperature, and that this relationship holds true over space and time (Deutsch et al., 2008). The validity of this assumption has not, to the best of our knowledge, been rigorously tested. This is a crucial aspect to understand and to incorporate into the growing body of literature supporting inclusion of mechanism into predictive models (Briscoe et al., 2023; Howard et al., 2023).

In predictive (correlative) models that do not capture the mechanisms by which species respond to climate, apparent associations between climate and distributions may be as much driven by the spatial structure of climate as by real biological process. Indeed, spurious pseudo‐predictors have been shown to perform just as well at predicting species distributions as a real set of climatic predictors (Fourcade et al., 2018). If air temperature does not relate to body temperature in a consistent way through time, and especially if these differences are biologically relevant, predictions of future species distributions based on present‐day correlations with air temperature will not be reliable.

Most organisms are ectotherms and thus have body temperatures that reflect their environment (Angilletta, 2009). Physiological functions are performed within a range of tolerable body temperatures, and critical functionalities such as locomotion, reproduction and growth are strongly related to body temperature. This means that ectotherm body temperature can be used to estimate its performance (the rate at which an organism can perform an ecologically relevant activity (Sinclair et al., 2016)) or fitness (Huey & Slatkin, 1976) in a specific climate and, by extension, to predict the ecological consequences for ectotherms of climate warming (e.g., Deutsch et al., 2008; Levy et al., 2015; Vasseuer et al., 2014).

It is critical to understand the relationship between ectotherm body temperature and air temperature when using only the latter to make these predictions, so that we may understand the impacts of climate change on the most numerous and arguably most important terrestrial animals. Indeed, given the fundamental role of ectotherms, particularly insects, in food webs and in providing ecosystem services, their loss due to climate warming will have impacts at organismal, and ecosystem levels as well as profound economic implications (Wagner, 2020).

There are reasons why body temperature may not scale predictably with air temperature. Organisms absorb radiation from sunlight and exchange heat with their immediate microenvironments via conduction, convection, and evaporative water loss (Campbell & Norman, 2000; Porter & Gates, 1969). These microenvironments are themselves governed by processes of heat exchange and this means that conditions near to the ground, where most terrestrial organisms live, are typically very different to air temperatures measured at 1.5–2 m above the ground (Lembrechts et al., 2019). These differences are in part determined by wind speed, relative humidity, and radiation; climate phenomena that, in addition to temperature increase, are known to be influenced by anthropogenic climate change (Cox et al., 2020).

Furthermore, many ectotherms use behavioural thermoregulation to manage their body temperature; for example, they may bask in the sun to warm up or seek shade to avoid overheating (Kearney et al., 2009). The potential surface activity times for an organism will be incorrectly estimated by air temperature if it does not capture the range of body temperatures that determine when and where activity can occur in the environment (Sears et al., 2011, 2019).

In this study we ask: (1) what is the relationship between body temperature and ambient air temperature?, (2) does this relationship vary across years?, (3) are differences between air and body temperature biologically relevant? And (4) to what extent does solar radiation drive differences between the body temperature and air temperature? We use field crickets (*Gryllus campestris*) in northern Spain as a model ectotherm species. *G. campestris* is a useful model species because it is a typical temperate insect, with an annual lifecycle and a window of tolerated temperatures. It uses behavioural thermoregulation (basking in sunshine and shade‐seeking) to manage its body temperature (Rodríguez‐Muñoz et al., 2023).

## 2 METHODS

### 2.1 Study system

We carried out fieldwork in the WildCrickets meadow in northern Spain (see www.wildcrickets.org). The meadow comprises homogeneous open short grassland (Supporting Information S1, Figure S1) and is home to a wild population of field crickets (*Gryllus campestris*). *G. campestris* inhabits grasslands throughout northern Europe (Panagiotopoulou et al., 2016) with late‐instar nymphs digging burrows in autumn and overwintering in them to complete development to adulthood in spring. Adults spend most of their time either in their burrow or in the immediate vicinity of their burrow where they position themselves on an area of ground cleared of most vegetation (Makai et al., 2020). The breeding season lasts for 2–3 months during which time all mating and egg laying occurs and adults die out in early to mid‐summer leaving their offspring to develop over the late summer. Here we restrict ourselves to consideration of the adult phase of the life cycle, which at our field site runs between May and July. The average temperature for these months is 16.4°C and the average total rainfall is 162.5 mm (mean values 2008–2020 recorded by the weather station positioned in the middle of the meadow). We consider a single life stage as different life stages contribute differentially to total lifetime fitness (Both & Visser, 2005; Crozier, 2003; Dempster, 1983; Kingsolver, 1989). The adult life stage is important as this is when reproduction occurs, and thus responses of sexually mature individuals to climate can have immediate effects on population dynamics and ecological processes (Khaliq et al., 2014).

### 2.2 Biophysical model

We constructed a biophysical model to predict cricket body temperature ($T_{b}$) from microenvironmental variables, namely solar radiation, air and ground temperature and wind speed, and the physical properties of the organism. This was a four‐step process that involved (1) sourcing long‐term (1985–2019) climate data for the WildCrickets meadow; (2) bias‐correcting these data against historic weather station data collected in the meadow over a shorter period (2010–2019); (3) running a microclimate model using the bias‐corrected data and (4) computing cricket body temperature using radiation, wind speed and temperature outputs from the microclimate model. These steps are explained in further detail below.

1. We sourced long‐term (1985–2019) climate data for the WildCrickets meadow using the mcera5 R package (Klinges et al., 2022). By default, mcera5 queries the ‘ERA5 hourly data on single levels from 1979 to present’ dataset (Copernicus Climate Change Services, 2017), which provides the most appropriate vertical and temporal resolutions for microclimate modelling. We built (‘build_era5_request’) and downloaded (‘request_era5’) climate data for the WildCrickets meadow. We then used the function ‘extract_clim’, to extract hourly values for air temperature (°C), specific humidity (kg/kg), pressure (Pa), windspeed (m/s), emissivity (downward long wave radiation flux divided by the sum of net long‐wave radiation flux and downward long wave radiation flux (unitless), cloud cover (per cent), direct normal irradiance (MJ/m^2^/h), and diffuse normal irradiance (MJ/m^2^/h). We used the function ‘extract_precip’ to extract daily precipitation (mm) values.
2. We corrected each climate variable against observed data collected by a weather station in the WildCrickets meadow following methods described in Maclean (2020). For each variable, we took ERA5 and weather station data for 2010–2019 and extracted 10,000 equally spaced values spanning the full range of both datasets. Coefficients were then derived by applying General Additive Models using the ‘gam’ function in the mgcv R package (Wood & Wood, 2015). The model was then applied to the ERA5 data for 1985–2019 to derive corrected values. For precipitation we also applied an additional ‘wet day’ adjustment as described in Maclean (2020). We calculated the ratio of observed weather station to ERA5 days with zero precipitation and adjusted the ERA5 data to give the expected proportion of days with zero precipitation by reducing precipitation on the days with the lowest rainfall to zero.
3. We used the corrected climate data to run a microclimate model for each year 1985–2019 and to derive air temperature, wind speed and below‐sward radiative fluxes for 0.01 m above the ground (approximately the height of the upper surface of an adult field cricket in its typical location on the ground outside a burrow). We ran the model using the ‘runwithNMR’ function in the microclimc R package (Maclean & Klinges, 2021). In the model, the vegetated surface is divided into multiple layers and the sward temperature of each layer is then calculated from the energy balance of that layer using the Penman–Monteith equation (Monteith, 1965; Penman, 1948). Ground heat fluxes are computed using the NicheMapR package (Kearney & Porter, 2017), which divides the soil layer into 20 layers and applies a set of simultaneous equations to solve for different vapour and heat fluxes between each layer, assuming a user‐specified soil type (sandy loam). Latent heat fluxes are computed by assuming that stomatal conductance follows a predictable relationship with photosynthetically active radiation absorption following Kelliher et al. (1995), with maximal stomatal conductance set at 0.33 mol m^−2^ s^−1^. Radiation was assumed to be attenuated by leaf area such that more radiation is absorbed in upper layers of the vegetated surface. Representation of the vertical distribution of leaf foliage density and area were derived using the ‘habitatvars’ function in the microctools package, specifying short grassland. This function returns typical latitude and seasonally dependent values for grassland derived from literature. The model then applies a set of simultaneous equations to account for heat exchange between leaves and air and between air layers within the grass surface to derive estimates of air temperature at the user‐specified height. Further details are provided in Maclean and Klinges (2021). Microclimate temperature estimates were validated against field measurements (see the Supporting Information S1).
4. We estimated cricket body temperature using a biophysical model based on heat exchange equations (for complete details, see the Supporting Information S1). In summary, the model calculates shortwave and longwave radiative gains, and thermal longwave, convective, and conductive heat losses, and predicts the equilibrium body temperature (*T*_b_) of a cricket, whereby the amount of energy absorbed equals the energy lost. This is made possible because components of the energy budget have a dependence on body temperature: for instance, if the cricket's body is much hotter than the air it loses heat more quickly. Thus, body temperature can be derived by equating the energy budget to zero and solving for temperature. Inputs to the model included total incoming shortwave radiation, air temperature, ground temperature and wind speed derived from the microclimate model, as well as organism‐specific parameters, including the mass, and solar absorptivity and emissivity. Body temperature estimates from the biophysical model were validated against field measurements of cricket body temperature (see Supporting Information S1).

We ran the biophysical model and computed cricket body temperature on an hourly time step for the months May–July (when adult crickets are active) for the years 1985–2019. We extracted predicted body temperature (hereafter body temperature) values for daytime hours (6 am to 7 pm).

### 2.3 Data analysis

To determine the relationship between hourly daytime (6 am to 7 pm) 2 m air temperature and body temperature values we ran a simple linear regression model.

To examine the inter‐annual variation in the relationship between air and body temperature we calculated three metrics for the months May–July for each year: (1) mean hourly temperature offset (body temperature minus air temperature), (2) body temperature degree hours above air temperature, (3) number of hours above 40°C for air temperature and body temperature. Metric (1) allows us to understand whether the difference between air and body temperature are constant (an assumption of models that use air temperature to predict species responses to climate change). Metrics (2) and (3) hold biological relevance. Body temperature degree hours indicate the extent to which an organism's body temperature remains higher than the air temperature, which may correspond to potential activity time and physiological functioning. 40°C is the body temperature at which crickets seek shade (in prep). The number of hours that body temperature is above 40°C is therefore representative of the number of hours in which behavioural thermoregulation (shade‐seeking) will be required, at the cost of other activities, such as foraging for food and mates.

Temperature offset showed a non‐linear (curvilinear) relationship with solar radiation and so we ran a linear regression including a quadratic term to investigate this relationship statistically.

All modelling and analyses were conducted using R statistical computing software Version 4.3.1 (R Core Team, 2023).

## 3 RESULTS

### 3.1 Model validation

The microclimate model reconstructed the hourly ground temperature in the meadow with a RMSE of 1.44°C (RMSE of 0.97°C for daily mean values; Supporting Information S1, Figure S2). The thermal model predicted cricket body temperature with a RMSE of 3.7°C (RMSE of 3.1°C for 20‐min mean values; Supporting Information S1, Figure S4 and Table S1).

### 3.2 The relationship between the body temperature and ambient air temperature

**FIGURE 1:**
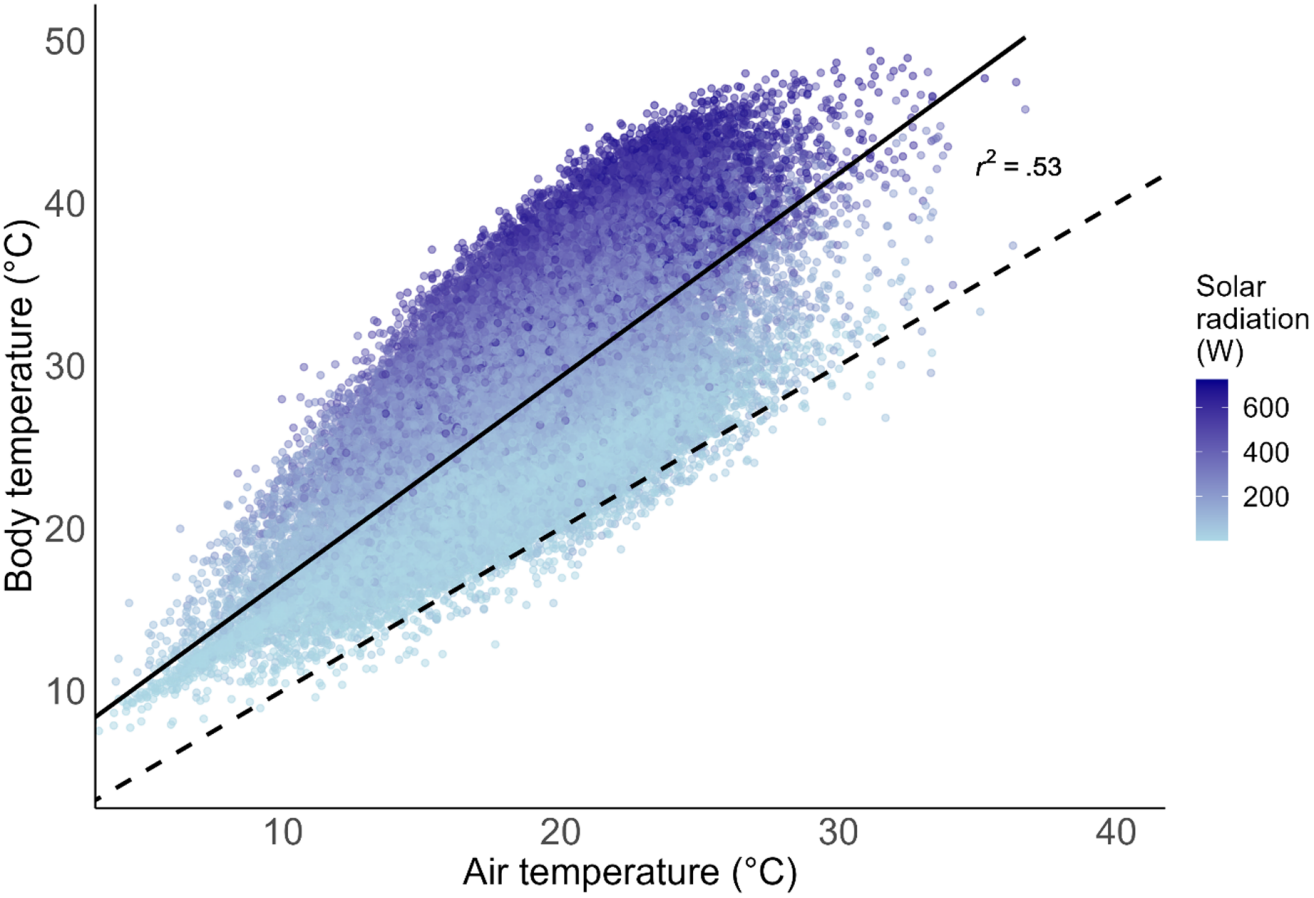
Linear regression showing the relationship between ambient air temperature and the predicted body temperature in *Gryllus campestris*. Data are mean hourly values from May–July for the years 1985–2019. Black solid line indicates the linear regression line, with the 95% confidence interval shown in grey. Dashed line indicates the 1:1 relationship. The *r* ^2^ value is .53.

Mean hourly body temperature was 27.4°C (range 7.5–49.4°C) and mean hourly air temperature was 18.5°C (range 3.0–36.7°C). Body temperature was positively correlated with air temperature (*r* ^2^ = .53, *F*_(1,45,078)_ = 50,760, *p* < .0001; Figure 1). Body temperature mostly exceeded air temperature (Figure 1). The difference between body temperature and air temperature (temperature offset) ranged from −4.8 to 23.6°C (mean difference 8.9°C).

**FIGURE 2:**
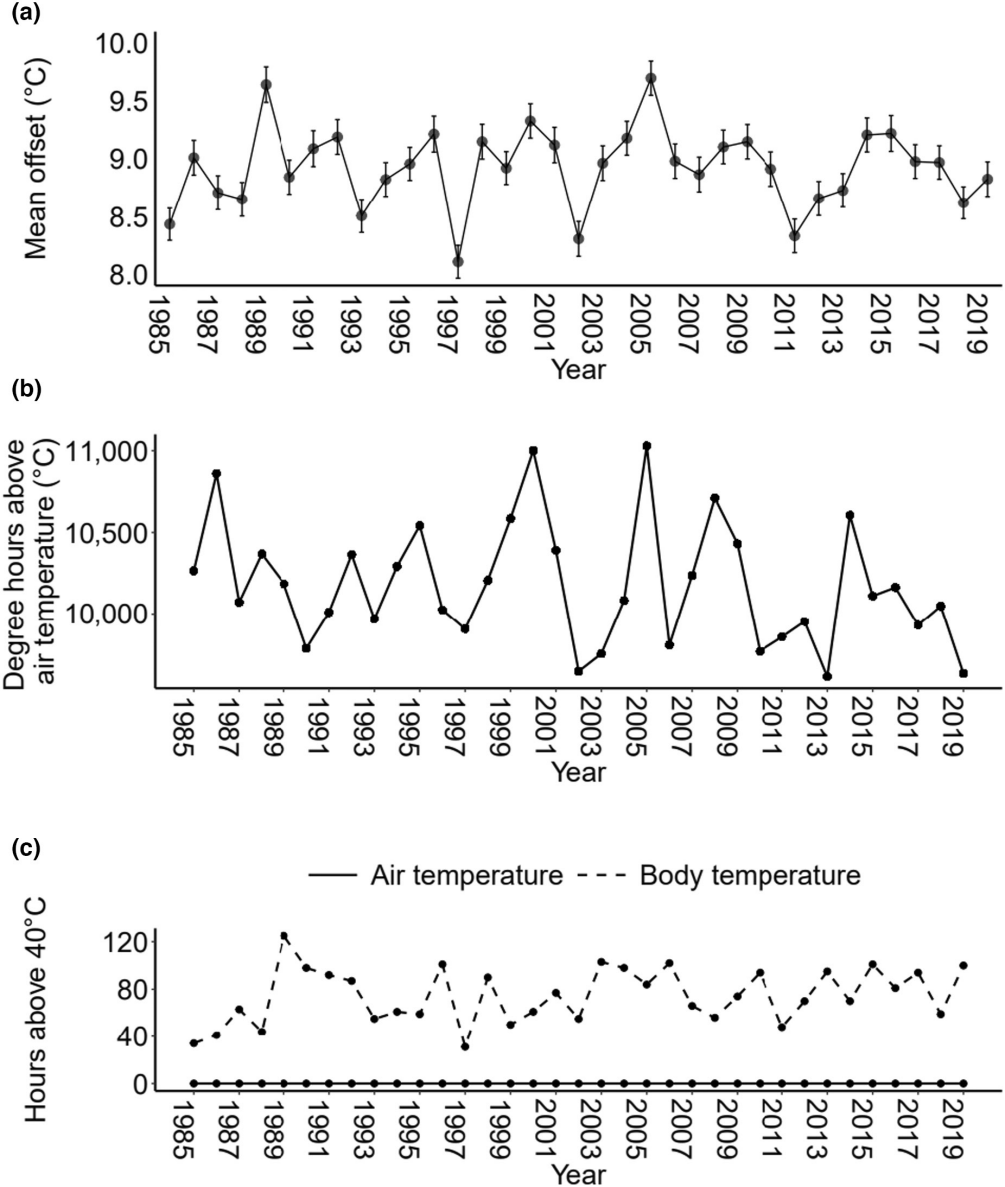
(a) mean temperature offset (°C) with standard error bars, (b) body temperature degree hours above air temperature (°C) and (c) number of hours above 40°C for air and body temperature. Data used are hourly daytime predicted body temperature and air temperature (°C) for May–July 1985–2019. Temperature offset was calculated as body temperature minus air temperature. Body temperatures above 40°C are not included in the degree hours calculation as the cricket will always seek shade.

The mean temperature offset for each year ranged from 8.1 to 9.7°C (Figure 2a). The cumulative number of degree hours above air temperature also varied between years and ranged from 9612 to 11,029°C•h (Figure 2b). The number of hours that body temperature exceeded 40°C ranged from 31 to 125 h, but was always greater than zero, whereas the air temperature never rose above 40°C (Figure 2c).

### 3.3 Solar radiation drives differences between the body temperature and air temperature

**FIGURE 3:**
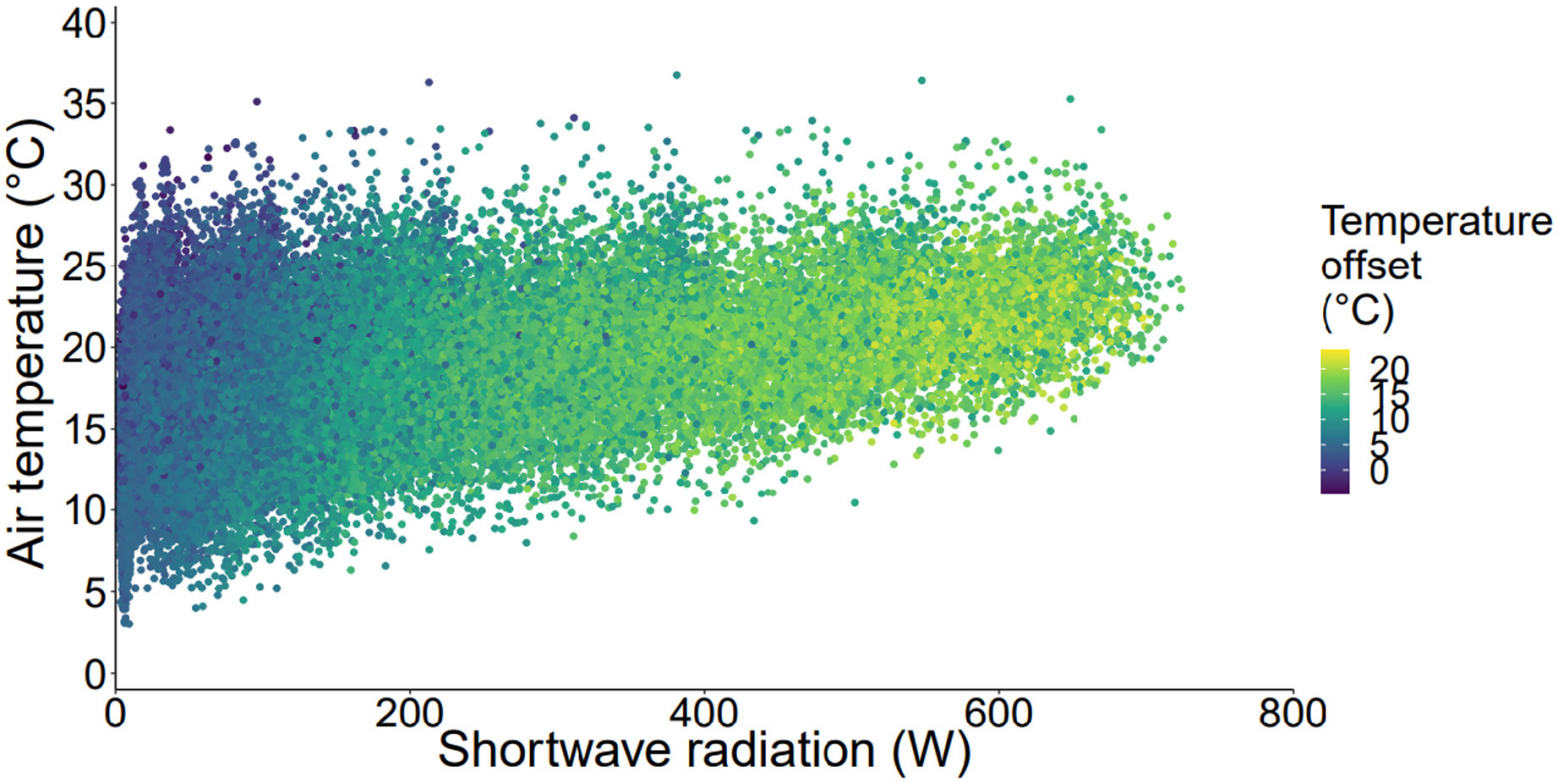
Temperature offset in relation to air temperature and solar radiation. Data used are hourly daytime predicted body temperature and air temperature and shortwave radiation for May–July 1985–2019. Temperature offset was calculated as body temperature minus air temperature.

There was a significant relationship between air temperature and temperature offset (body temperature minus air temperature), but only 4.3% of the variance in temperature offset could be explained by changes in air temperature (*F*_(1,45,078)_ = 2041, *r* ^2^ = .043, *p* < .001). Temperature offset showed a strong, quadratic relationship with solar radiation (*F*_1,45,077_ = 109,800, *r* ^2^ = .83, *p* = <.001). At lower levels of solar radiation, as solar radiation increased, temperature offset rose at an increasing rate. However, this increase slowed down and began to plateau at higher levels of solar radiation (from approximately 400 W; Figure 3).

## 4 DISCUSSION

Air temperature is often used to model species distributions and predict their responses to climate change (Gardner et al., 2019). However, when applied to animals, these models rely on the assumption that air temperature is an effective proxy for body temperature, which ultimately determines thermal performance. Here we show that body temperature of an ectotherm is not reliably predictable from air temperature alone. We find that the body temperature is usually higher than air temperature and while there is a positive relationship between air and body temperature, it is confounded by the additional influence of radiation. Notably, when solar radiation is low, body temperature can drop below air temperature owing to the emittance of thermal radiation, and when solar radiation is high, body temperature can rise to values more than 20°C above the air temperature.

An important finding is that differences between air and body temperature are not consistent across different years. This highlights that the relationship between air and body temperature cannot be assumed to remain constant through time, which is a critical implicit assumption of species distribution models (SDMs) that are constructed using ambient temperatures and extrapolated into the future under climate change. Thus, species may be unexpectedly present or absent in certain locations due to inaccuracies in estimating their body temperature within the model.

We also show that the differences between air and body temperature are biological relevant. For example, year‐to‐year variability in cumulative degree hours above air temperature for body temperature means that air temperature alone does not capture the breadth and dynamics of a species' body temperature fluctuations. Body temperature impacts the physiological performance of ectotherms, and metabolic rates, digestion, growth, reproduction, and overall activity levels are closely tied to body temperature. Consequently, deviations in body temperature from the ambient air temperature means that air temperature alone cannot capture when and where activity can occur in the environment.

Air temperature also missed the extent to which body temperature exceeded 40°C. This could mislead assessments of species' vulnerability to climate change for two reasons. Firstly, air temperature will not reflect the necessity for thermoregulatory behaviour at certain body temperatures. Behavioural thermoregulation (shade seeking) could allow species to persist in a warming world (Sears et al., 2016), although it is also likely to be costly (for example, spending more time hiding from the sun can reduce time available for foraging for food and mates). Secondly, species will have critical maximum temperature thresholds for their physiological functioning. Using air temperature as a proxy for body temperature could suggest species are operating well below this maximum and have a high ‘thermal safety margin’ (the difference between a species' maximum tolerance to heat and the air temperature of its environment) and the ability to tolerate warming conditions. However, these thermal safety margins are likely to be overestimated if body temperature is in fact much higher than air temperature. Species could already be close to thermal maximum limits on a clear, sunny day (Sunday et al., 2014) and in consequence, could be less able to tolerate warming and more vulnerable to climate change than would be predicted by air temperature. This problem is likely to be particularly relevant to species in sunny environments because they may already be living close to their optimal temperature and so increases in body temperature will have the most deleterious consequences (Deutsch et al., 2008).

We show that solar radiation has an important effect in driving differences between air and body temperature. This is relevant, because the space‐for‐time substitution implicit in correlative SDMs assumes that relationships between body and ambient temperature that exist in space also persist through time, and yet radiation varies spatially. Furthermore, climate change is expected to alter multiple aspects of weather, not just air temperature (IPCC, 2013). For example, Cox et al. (2020) found that regions that have undergone an increase of >0.5°C more warming during the daytime than the night‐time have experienced increased levels of cloud cover. Cloud attenuates shortwave radiation and would reduce the radiation absorbed by a species and limit body temperature, even if air temperature is high. Cox et al. (2020) found that in other places daytime cloud cover has reduced. In these areas, warmer air temperatures and higher solar insolation will have thermal consequences for ectotherms that are much greater than expected by air temperature alone. Given the complex determinants of body temperature, any current correlations between air and body temperature cannot be extrapolated reliably into the future (Sinclair et al., 2016); climate change could make the present‐day deviations between air temperature and body temperature even more pronounced and so make it increasingly likely for current relationships between air temperature and body temperature to change.

Behaviour will influence the environment that an organism experiences (Angilletta, 2009; Huey, 1991) and thus complicate the translation between environmental and body temperatures. Many ectotherms use thermoregulatory behaviour to maintain favourable body temperature (for example, seeking or retreating from direct or indirect solar radiation (Cossins & Bowler, 1987)). Stevenson (1985), for example, calculated that through behavioural thermoregulatory mechanisms (changing activity time or microhabitat selection), a 1 kg ectotherm could modify its body temperature by up to 45°C. The greater surface area to volume ratio of small ectotherms means that behaviour has the potential to be even more effective as a method for regulating temperature. Although our model does not include behaviour, body temperature estimates can imply potential activity times and the extent to which thermoregulatory behaviour (e.g., shade seeking) may be required.

There will inevitably be a limit to the extent that an organism can use behaviour to optimise its temperature and buffer itself against negative impacts of climate change (Kearney et al., 2009). Our model is a fundamental component to fully mechanistic models for ectotherms that consider both thermo‐ecophysiology and the expression of behavioural thermoregulation in the wild. Adding a behavioural component to our biophysical model is the next step for our research.

Our model is transferable to other ectotherms, with adjusted morphologically based parameters. Although the model makes some assumptions about organism shape (here approximating the cricket as an ellipsoid), this allows the inputs to the model to remain relatively simple (mass). Although a more complicated model may estimate body temperature with a higher degree of accuracy, this is traded off with the requirement for more information on the organism, which may not be available or easily obtainable. Our model can, however, be run for any location, because the climate data required to run microclimate models are available for the globe, and for past and future climates (Lembrechts & Lenoir, 2020; Maclean, 2020).

## 5 CONCLUSION

Global temperatures will continue to rise due to anthropogenic climate change. To predict the consequences for biodiversity requires an understanding of how body temperature drives patterns of species distributions and the relationship between the changing temperature of the environment and body temperature. The absorption of solar radiation, convection and conduction are important mechanisms that can cause the body temperature of an ectotherm to diverge from the surrounding air temperature and so it is not sufficient to estimate body temperature based on air temperature alone. SDMs have been applied to assess species' extinction risk (Thomas et al., 2004), set conservation priorities (Moradi et al., 2019), consider the design of protected area networks (Araújo et al., 2011) and to estimate the risk of future crop losses (Beck, 2013). This practical importance of SDMs makes it crucial for their results to be reliable. Models that consider the mechanisms that underlie the biological processes by which species respond to climate change could help to inform better conservation decisions in a changing climate.

## AUTHOR CONTRIBUTIONS

**Alexandra S. Gardner:** Conceptualization (equal); formal analysis (equal); investigation (equal); methodology (equal); validation (equal); writing – original draft (equal); writing – review and editing (equal). **Ilya M. D. Maclean:** Conceptualization (equal); funding acquisition (equal); methodology (equal); project administration (equal); supervision (equal); writing – review and editing (equal). **Rolando Rodríguez‐Muñoz:** Data curation (equal); methodology (equal); writing – review and editing (equal). **Paul E. Hopwood:** Data curation (equal); writing – review and editing (equal). **Kali Mills:** Data curation (equal); investigation (equal); methodology (equal); writing – review and editing (equal). **Ross Wotherspoon:** Data curation (equal); investigation (equal); methodology (equal); writing – review and editing (equal). **Tom Tregenza:** Conceptualization (equal); funding acquisition (equal); methodology (equal); project administration (equal); resources (equal); supervision (equal); writing – review and editing (equal).

## FUNDING INFORMATION

This work was funded by the Natural Environment Research Council (NERC) [Grant Number: NE/V000772/1].

## CONFLICT OF INTEREST STATEMENT

The authors declare that there are no conflicts of interest.

## Supporting information


Data S1

## Data Availability

The data and code supporting this manuscript are available in Zenodo: DOI 10.5281/zenodo.8383661.
