# Supplementary material for: The relationship between the body and air temperature in a terrestrial ectotherm
Source: Ecol Evol. 2024 Feb 13;14(2):e11019. doi: 10.1002/ece3.11019 (PMC10862186; doi:10.1002/ece3.11019)
Supplement: Supplementary file 1 — Data S1 [file ECE3-14-e11019-s001.docx]

**Supporting Information**


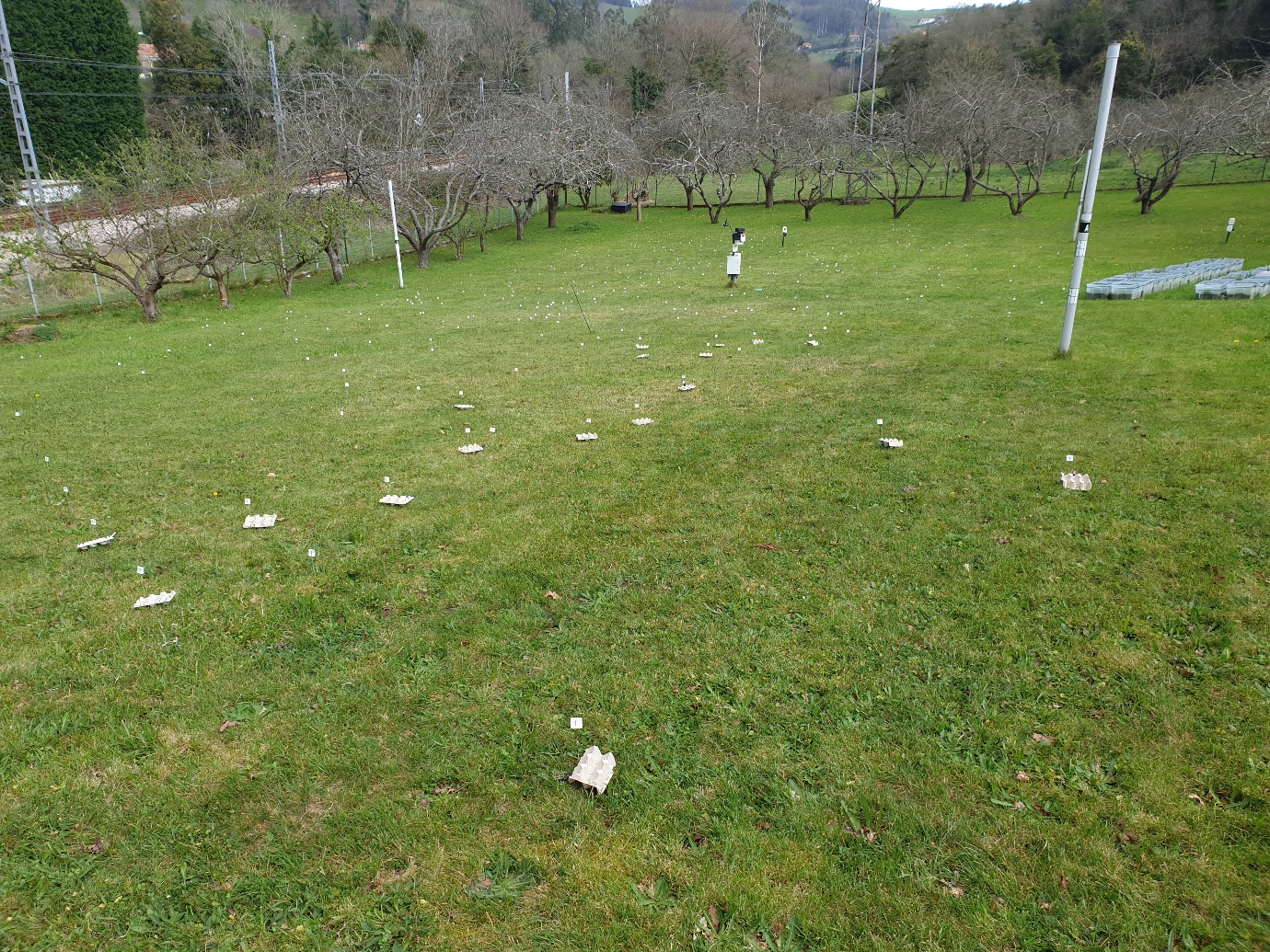


**Figure S1: The WildCrickets meadow**

**Biophysical model**

$T_{b}$ calculations consider the organism’s energy budget. Heat gains and losses at the surface of the animal can be summarised as follows (Gates, 1980):

$$Q_{net}=Q_{sabs}+Q_{labs}-Q_{emit}-Q_{conv}-Q_{cond}-Q_{evap}+Q_{met}$$

Eq. 1

where $Q_{net}$ is the net energy exchange (W) with the environment, $Q_{sabs}$ is shortwave radiation absorbed, $Q_{labs}$ is longwave radiation absorbed, $Q_{emit}$ is thermal radiation emitted, $Q_{conv}$ is energy exchange due to convection and $Q_{cond}$ is energy exchange due to conduction, $Q_{evap}$ is energy emitted due to evaporative water loss and $Q_{met}$ is energy gained from metabolism.

These terms have a dependence on $T_{b}$ and the equilibrium body temperature can be determined by ensuring incoming energy matches outgoing energy. In other words, the amount of energy absorbed equals the energy lost. We considered evaporative and metabolic heat exchange to be negligible for crickets (like in other insects e.g., Anderson et al., 1979; Buckley et al., 2013) and solved the energy balance to determine cricket $T_{b}$ at hourly intervals as:

$$Q_{sabs}+Q_{labs}-Q_{emit}-Q_{conv}-Q_{cond}=0$$

Eq. 2

$T_{b}$ was solved for by finding the root of Eq. 2 using the ‘uniroot’ function of R base packages (R Core Team, 2023), which when applied in this context iteratively finds the body temperature that equates Eq. 2 to zero.

We describe how the components of Eq. 2 were calculated below, but we follow calculations of the steady state heat budget model for ectotherms developed by Kearney and Porter (2020). We assumed a cricket mass of 1.3g (mean of all weights taken prior to 73 field trials, details below) and density of 1000kg/m^3^ (Kearney & Porter, 2020).

*Shortwave radiation absorbed (*$Q_{sabs}$*)*

We calculated absorbed shortwave radiation considering the proportion of the cricket exposed to direct normal, diffuse and reflected components of incoming solar radiation, as follows:

$$Q_{sabs}=a\left( A_{dir}\cdot S_{dir}+A_{sky}\cdot S_{dif}+A_{ground}\cdot S_{ref} \right)$$

Eq. 3

where $a$ is cricket solar absorptivity (set at 0.7 (Anderson et al., 1979)), $A_{sky}$ and $A_{ground}$ are the surface areas exposed to diffuse, and reflected solar radiation, respectively (both set at 0.5*A*, where *A* is the total cricket surface area), $S_{dir}$, $S_{dif}$ and $S_{ref}$are the flux density of incident direct normal, diffuse and reflected solar radiation, respectively and $A_{dir}$ is the silhouetted area of the cricket normal to the solar beam (0.245A), calculated by assuming the cricket to be triaxial ellipsoid-shaped (see Kearney & Porter, 2020).

*Longwave radiation absorbed (*$Q_{labs}$*)*

We calculated absorbed longwave (emitted from the sky and ground) radiation as:

$$Q_{labs}=A_{sky}\cdot\varepsilon\cdot\sigma\cdot\left( {T_{a}}^{4} \right) +A_{ground}\cdot\varepsilon\cdot\sigma\cdot\left( {T_{g}}^{4} \right)$$

Eq. 4

Where $\varepsilon$ is cricket emissivity to thermal radiation, $\sigma$ is the Stefan Boltzman constant (5.673 x 10^-8^ Wm^−2^K^−4^), $T_{a}$ is microclimate air temperature in Kelvin and $T_{g}$ is ground temperature in Kelvin.

$\varepsilon$ was set to 0.95, which we determined by measurements in the field using a FLIR T440 model infra-red camera (FLIR Systems Inc., http://www.flir.com/instruments/display/?id=62960).

*Thermal radiation emitted (*$Q_{emit}$*)*

Heat is lost through longwave radiation emitted by the cricket (Porter & Gates, 1969). The total energy emitted by the cricket is proportional to the fourth power of its body temperature:

$$Q_{emit}=A\cdot\varepsilon\cdot\sigma\cdot\left( {T_{b}}^{4} \right)$$

Eq. 5

Where $A$ is the total cricket surface area and $T_{b}$ is body temperature in Kelvin.

*Energy exchange due to convection (*$Q_{conv}$*)*

Heat is lost due to convection, with the rate of heat transfer per unit body area given by the Newton law of cooling:

$$Q_{conv}={A\cdot h}_{c}\cdot\left( T_{b} - T_{a} \right)$$

Eq. 6

Where $h_{c}$ is the convective heat transfer coefficient (W m^-2^K^-1^).

$h_{c}$ is specific to the size and shape of an organism. Again, we approximated the cricket as a triaxial ellipsoid shape and calculated $h_{c}$ for mixed (free and forced) convection using dimensionless numbers (Nusselt (${{Nu}_{total}, Nu}_{free}$, ${Nu}_{forced}$), Rayleigh ($R_{a}$), Grashof ($G_{r}$), Prandtl ($P_{r}$) and Reynolds ($R_{e}$)) as follows:

$$h_{c}= {Nu}_{total}\cdot\left( \frac{K_{a}}{D} \right)$$

Eq. 7

Where ${Nu}_{total}$ is the Nusselt number for free and forced convection, $K_{a}$ is the thermal conductivity of air (W·m^-1·^K^-1^) and $D$ is the characteristic dimension and equal to the cube root of cricket volume (Mitchell, 1976), which in turn was calculated as mass (in kg) divided by density (1000 kg/m^3^ (Kearney & Porter, 2020).

${Nu}_{total}$ is obtained using an empirical combining rule:

$${Nu}_{total}=({{Nu}_{free}}^{3}+ {{{Nu}_{forced}}^{3})}^{\frac{1}{3}}$$

Eq. 8

Where ${Nu}_{free}$ is the Nusselt number for free convection calculated as:

$${Nu}_{free}=2+0.6\cdot R_{a}$$

Eq. 9

And where ${Nu}_{forced}$ is the Nusselt number for forced convection calculated as:

$${Nu}_{forced}=0.35\cdot{R_{e}}^{0.6}$$

Eq. 10

The Rayleigh number ($R_{a}$) is calculated as:

$$R_{a}={G_{r}}^{\frac{1}{4}}\cdot{P_{r}}^{\frac{1}{3}}$$

Eq. 11

The Grashof number ($G_{r}$) is calculated as:

$$G_{r}=|(\rho^{2}\cdot(1-T_{a})\cdot g\cdot D^{3} \cdot\frac{\left( T_{b} - T_{a} \right)}{\mu^{2}})|$$

Eq. 12

Where $\rho$ is the air density (kg·m^-3^), $g$ is the gravitational constant (9.81 ms^2^) and $\mu$ is dynamic viscosity (kg·m^-1^s^-1^).

The Prandtl number ($P_{r}$) is calculated as:

$$P_{r}=c_{p}\cdot\mu/K_{a}$$

Eq. 13

Where $c_{p}$ is the specific heat at constant pressure (1005.7 J·kg^-1^K^-1^).

The Reynolds number ($R_{e}$) is calculated as:

$$R_{e}=\rho\cdot V\cdot D/\mu$$

Eq. 14

Where $V$ is windspeed (ms^-1^).

For Eqs. 12, 13 and 14, $\mu$, and $K_{a}$were calculated using the ‘DRYAIR’ function in the NicheMapR package (Kearney & Porter, 2017).

*Energy exchange due to conduction (*$Q_{cond}$*)*

Heat exchange due to conduction ($Q_{cond}$) depends on the temperature difference between the body and the ground:

$$Q_{cond}=A_{contact}\cdot K_{o} \cdot\left( T_{b} - T_{g} \right)$$

Eq. 15

Where $A_{contact}$ is the surface area of the cricket in contact with the ground (m^2^), set as 1% of the cricket total surface area (just the feet), and $K_{o}$ _=_ 15, which is the thermal conductance of the cricket (Wm^-2^K^-1^) derived assuming a conductivity of 0.15 Wm^-1^K^-1^ for insect cuticle with a thickness of 0.01m (Galushko et al., 2005).

**Model and method validations**

Microclimate model

We validated ground temperature estimates against values recorded in the meadow during May-July 2017 (when field trials for biophysical model validation took place) by a Vantage Pro2 weather station with a stainless-steel temperature probe attachment to measure surface air temperature (<https://www.davisinstruments.com/collections/add-on-sensors/products/stainless-steel-temperature-probe-with-rj-connector>), both of Davis Instruments. The temperature probe is positioned on the ground. It is protected from the sun and wind by the surface vegetation (grass), which is mown throughout the season. We tested how well the microclimate model predicted the observed ground temperature by calculating the root mean square error (RMSE) (Piñeiro et al., 2008).


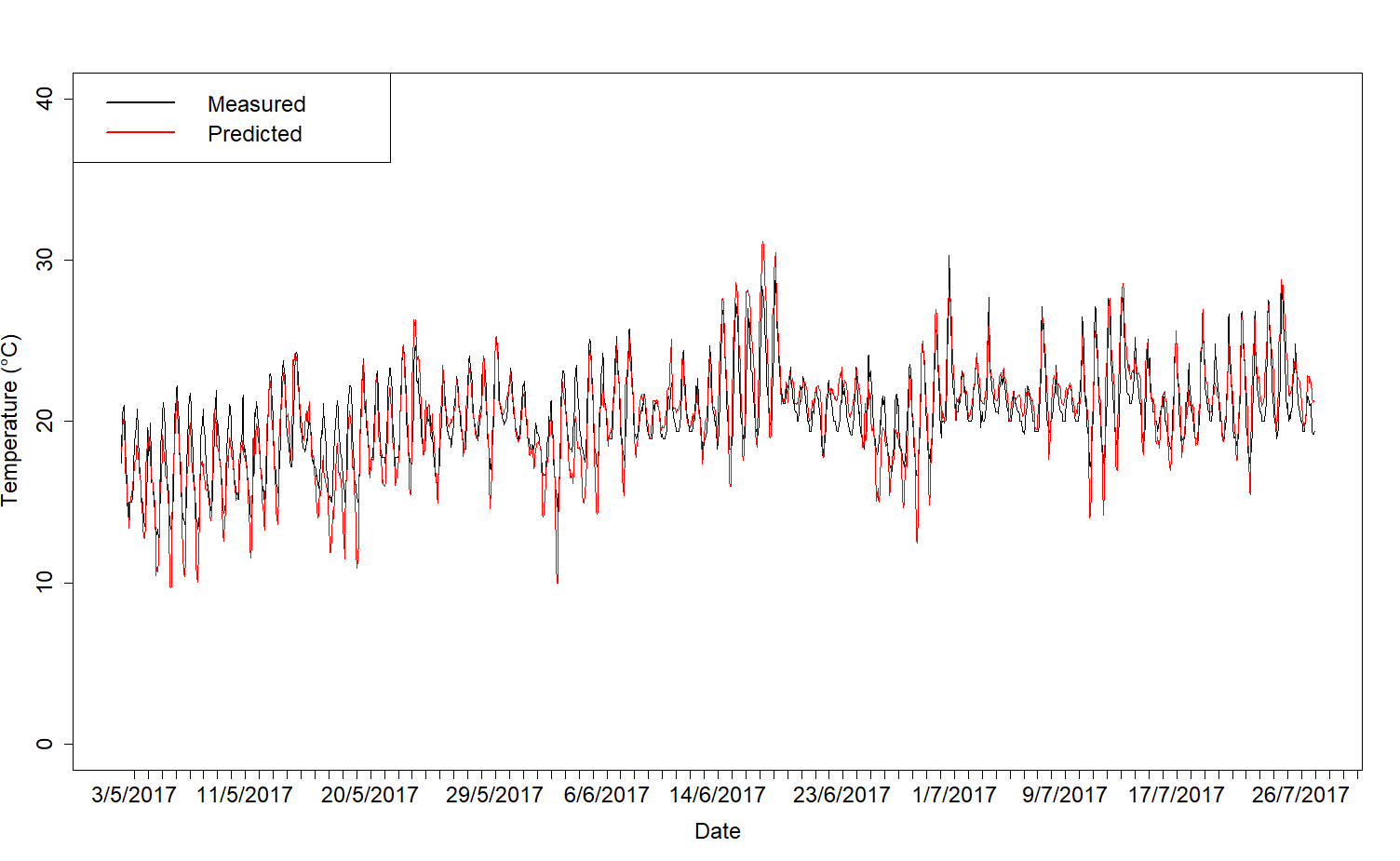


**Figure S2: Predicted and observed ground temperature.** Data are for May-July 2017. The root mean square error (RMSE) for hourly values is 1.44°C. The RMSE for daily mean temperatures is 0.97°C.

Biophysical model

We validated the biophysical model using measurements of body temperature obtained in the field. Field crickets (*Gryllus campestris*) were collected from the WildCrickets meadow in La Aguda, Asturias, Spain in May 2017. The weight of each cricket was taken and recorded, before the thorax was sanded down with a razor blade and tagged with a 2 letter or 1 letter/1 number combination for identification using superglue (Figure S3).

**Figure S3: Basking adults with identification tags visible.**

A copper loop was superglued to the tag so that during the heating trials, a piece of wire could be passed through the copper loop to make a lead. The crickets were housed in a temperature-monitored room with food, water and with lighting set at the natural day/night cycle to match the cricket’s circadian rhythm from May-July.

At the start of each heating trial, crickets were placed in a cool box until they reached a temperature below 13°C. 13°C was chosen as it was deemed to be below the likely body temperature a cricket would experience naturally in the wild, whilst remaining nonlethal, allowing us to capture the full range of temperatures that a cricket would experience.

Crickets were then placed outside in a trial ‘arena’ - a 2x3m square of mown grass in the WildCricket meadow. The cricket’s lead was attached to the ground to restrict cricket movement during the trial. Cricket body temperature was recorded every 20 seconds for 20 minutes using a Flir T440 infra-red camera. Trials took place in all dry weather conditions.

We conducted 73 trials with live crickets. Each cricket was cooled to 13°C then anchored to a patch of short grass with a copper lead. A calibrated FLIR T440 model infrared camera (FLIR Systems Inc., http://www.flir.com/instruments/display/?id=62960) was used to record cricket temperature every 20 seconds for 20 minutes.

Body temperature measurements were taken from the head and assumed to be the temperature of the whole cricket as in Remmert (1985). Ambient conditions (solar radiation, 0.01m air temperature, ground temperature) were recorded during the trials and used as inputs to the biophysical model.

As cricket body temperature at the start of the trial was known, we could run the biophysical model as a transient model and derive body temperature at each subsequent time step. We assumed cricket thermal capacity as 3.5 Jg^-1^K^-1^ (Wang et al., 2003).

We tested how well the biophysical model predicted the observed cricket body temperature by calculating the RMSE. We also compared the model residual with solar radiation to detect variability in model performance under different environmental conditions and with cricket mass and sex to detect variability in the performance of the model with different individuals.

Cricket body temperature increased during the heating trials in a curvilinear fashion. Cricket body temperature reached equilibrium with the environment within 10-15 minutes from the start of each trial (e.g., Figure S4a).

We plotted observed and predicted cricket body temperature values on the same y-axis (Figure S4a-b). Across all trials the mean RMSE was 3.63°C (range 0.42-8.11°C) (Table S1). The accuracy of the biophysical model did not vary with solar radiation and was not affected by the sex or mass of the cricket (Figures S5-7).


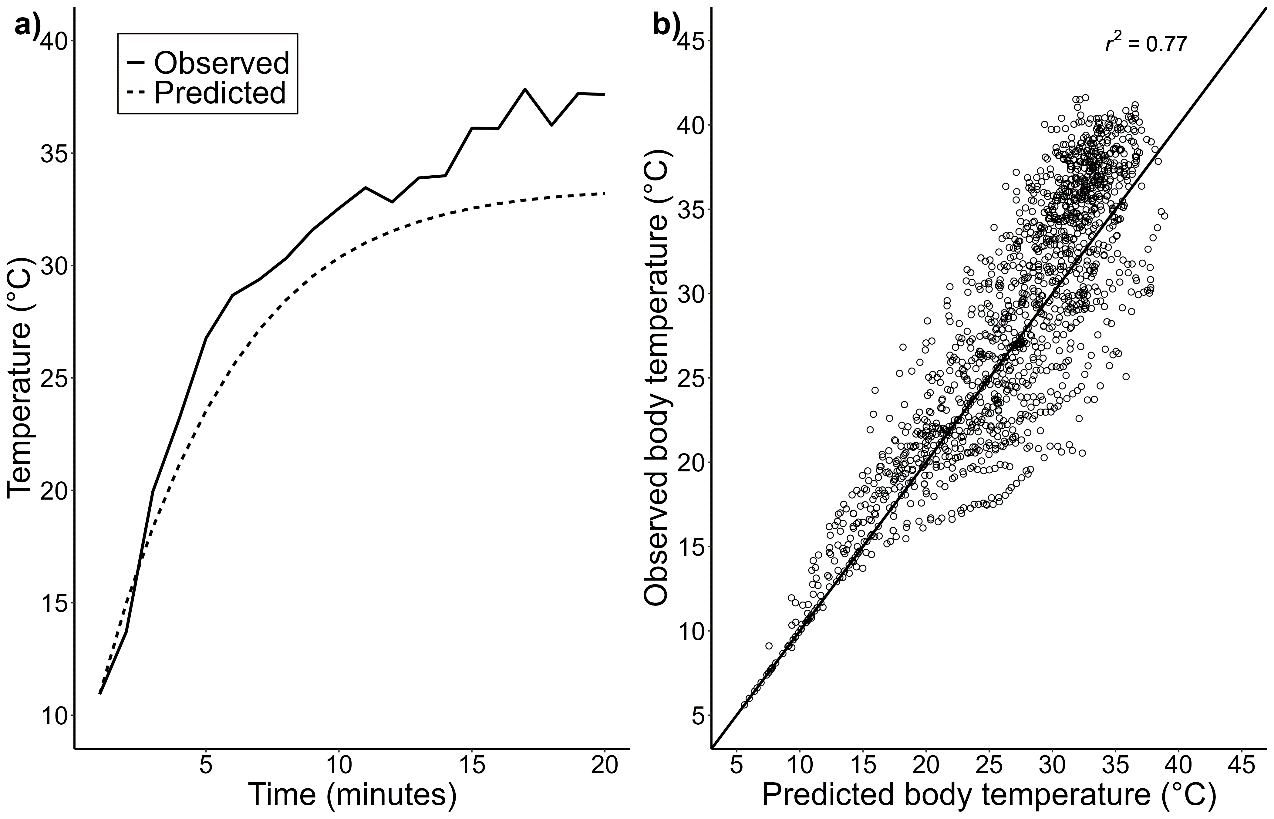


**Figure S4: Observed and predicted cricket body temperature.** a) data for a single heating trial for a male cricket weighing 0.95g. In this example, the mean RMSE is 2.82°C. RMSE for all 73 trials are presented in Table S1. b) the correlation between predicted and observed body temperature. Data are predicted and observed body temperature for each minute across all trials (73 x 20 minutes). The solid black line indicates the linear (1:1) relationship. r^2^=0.77.

**Table S1: Mean observed and predicted body temperature (**$\boldsymbol{T}_{\boldsymbol{b}}$**) and RMSE for each trial (n=73).**

| **Trial** | **Mean observed Tb** | **Mean predicted Tb** | **Trial RMSE** |
| --- | --- | --- | --- |
| 1 | 29.02 | 28.47 | 1.14 |
| 2 | 31.7 | 29.99 | 1.87 |
| 3 | 32.03 | 32.36 | 0.8 |
| 4 | 32.43 | 31.03 | 1.58 |
| 5 | 33.68 | 31.42 | 2.68 |
| 6 | 35.42 | 31.1 | 4.61 |
| 7 | 33.71 | 30.9 | 3.23 |
| 8 | 27.84 | 30.43 | 2.92 |
| 9 | 36.14 | 30.63 | 5.86 |
| 10 | 33.77 | 29.02 | 4.92 |
| 11 | 27.99 | 27.82 | 0.56 |
| 12 | 20.88 | 28.5 | 8.15 |
| 13 | 28.93 | 32.61 | 4.13 |
| 14 | 33.8 | 32.34 | 1.67 |
| 15 | 33.52 | 31.02 | 2.67 |
| 16 | 35.75 | 32.76 | 3.21 |
| 17 | 20.68 | 23.41 | 3.25 |
| 18 | 28.91 | 28.78 | 1.32 |
| 19 | 32.33 | 28.57 | 4.07 |
| 20 | 30.14 | 27.63 | 3.09 |
| 21 | 29.53 | 26.96 | 2.72 |
| 22 | 30.74 | 30.78 | 1.85 |
| 23 | 30.56 | 29.02 | 2.16 |
| 24 | 15.19 | 21.08 | 7.08 |
| 25 | 16.5 | 23.22 | 7.4 |
| 26 | 32.88 | 29.4 | 3.96 |
| 27 | 18.98 | 19.62 | 1.4 |
| 28 | 19.52 | 20.07 | 0.76 |
| 29 | 18.59 | 22.44 | 4.77 |
| 30 | 19.31 | 23.93 | 5.22 |
| 31 | 20.06 | 24.04 | 4.75 |
| 32 | 27.04 | 29.51 | 3.8 |
| 33 | 20.56 | 22.1 | 1.89 |
| 34 | 19.36 | 21.47 | 2.85 |
| 35 | 20.58 | 26.12 | 6.27 |
| 36 | 20.98 | 26.24 | 5.83 |
| 37 | 26.65 | 28.61 | 3.27 |
| 38 | 16.82 | 21.23 | 4.81 |
| 39 | 17.86 | 22.05 | 4.65 |
| 40 | 22.68 | 26.04 | 3.94 |
| 41 | 22.28 | 22.86 | 1.33 |
| 42 | 22.02 | 24.86 | 3.64 |
| 43 | 18.86 | 24.59 | 7.24 |
| 44 | 23.79 | 29.63 | 6.33 |
| 45 | 31.25 | 27.95 | 3.73 |
| 46 | 20.9 | 24.53 | 3.94 |
| 47 | 20.83 | 27.69 | 7.45 |
| 48 | 26.93 | 31.94 | 5.81 |
| 49 | 36.14 | 30.92 | 5.52 |
| 50 | 36.21 | 31.31 | 5.44 |
| 51 | 34.12 | 29.88 | 4.69 |
| 52 | 34.37 | 30.03 | 4.82 |
| 53 | 25.22 | 26.88 | 2.01 |
| 54 | 27.9 | 28.63 | 1.27 |
| 55 | 26.6 | 26.74 | 0.54 |
| 56 | 30.58 | 30.61 | 1.86 |
| 57 | 36.07 | 32.79 | 3.6 |
| 58 | 35.99 | 33.34 | 3 |
| 59 | 35.78 | 32.2 | 3.92 |
| 60 | 37.43 | 31.52 | 6.21 |
| 61 | 36.98 | 29.53 | 7.98 |
| 62 | 27.76 | 27.9 | 1 |
| 63 | 29.1 | 30.55 | 1.8 |
| 64 | 31.71 | 30.58 | 1.71 |
| 65 | 28.39 | 30.66 | 2.77 |
| 66 | 33.9 | 31.64 | 2.48 |
| 67 | 34.27 | 32.12 | 2.56 |
| 68 | 23.62 | 26.75 | 3.59 |
| 69 | 31.16 | 27.93 | 3.63 |
| 70 | 31.88 | 28.72 | 3.56 |
| 71 | 32.77 | 28.87 | 4.22 |
| 72 | 31.78 | 27.16 | 4.89 |
| 73 | 34.51 | 31.65 | 3.23 |


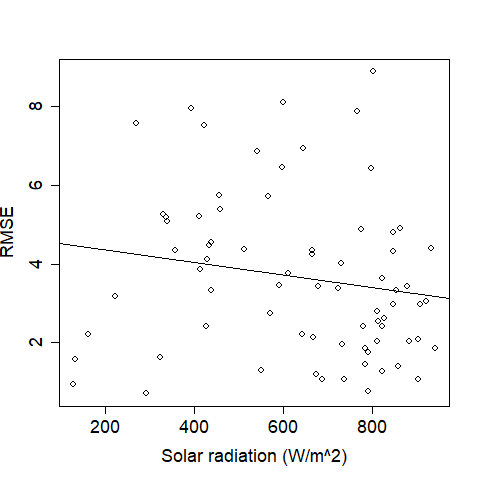


**Figure S5: Biophysical model RMSE at different values of mean solar radiation.** Data are for all trials. RMSE did not vary significantly with solar radiation (linear regression, F_1,71_=2.18, p=0.15, R^2^ = 0.030).


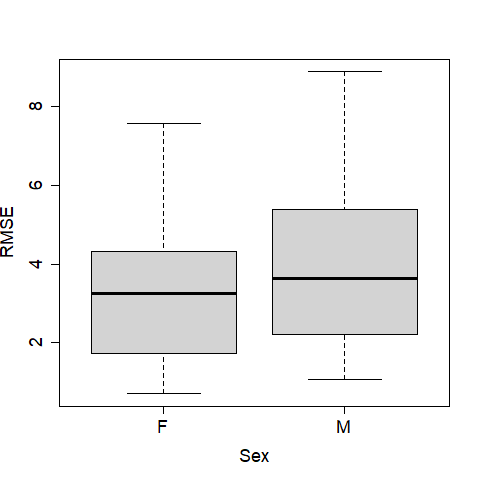


**Figure S6: Biophysical model RMSE for female (F) and male(M) crickets.** Data are for all trials. We found no significant difference between RMSE for male and female crickets (two sample t-test, t(71)=-1.80, p=0.17).


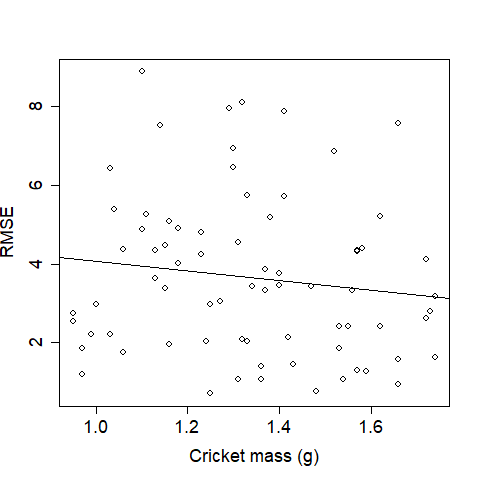


**Figure S7: Biophysical model RMSE at different cricket masses.** Data are for all trials. RMSE did not vary significantly with cricket mass (linear regression, F_1,71_=1.28, p=0.26, R^2^ = 0.018).

Thermal imaging camera temperature reading

We assessed the accuracy of the thermal imaging camera against a thermocouple (SurveyTag Microclimate Logger MC-01 with 0.075mm K-type thermocouple) as follows. A moribund adult cricket was placed in direct sunlight with the thermal camera positioned above using a tripod. On the thermal imaging camera interface, we positioned three temperature-recording ‘spots’ on the head, thorax and abdomen of the cricket (an example of a ‘spot’ can be seen in Figure S8).


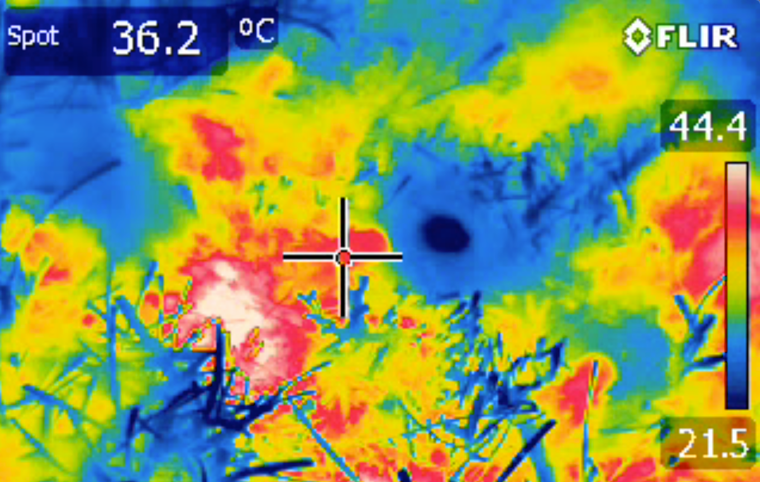


**Figure S8: The interface of the FLIR T440 Thermal Imaging Camera** showing a ‘spot’ positioned on the thorax of an adult cricket basking outside of a burrow. The ‘spot’ temperature is displayed in the top-left corner and the temperature range presented as a colour gradient is shown on the right side of the interface.

Two thermocouples were placed on the cricket, one on the abdomen and one on the thorax. The thermocouples were set to record body temperature every 30 seconds. The averaging value was set to 1 and burst length was set to 3000 milliseconds. This means that for each recorded temperature, 30 samples were collected every 100 milliseconds and averaged to produce one temperature reading every 30 seconds. For the thermal imaging camera, a photograph was taken every 30 seconds, mirroring the time intervals of the thermocouples and the temperature from each spot reading was collected. Temperature readings were collected for a total of 2 hours and 20 minutes and two bouts of artificial shade were introduced during this time, each lasting for 20 minutes. This was done to assess whether the two methods for measuring body temperature remained similar when the amount of solar radiation and thus the body temperature of the crickets fluctuated. To assess the similarity between the thermocouple measurements and the thermal camera measurements we calculated the RMSE (Piñeiro et al., 2008). The thermocouple and thermal imaging camera measurements were similar (Figure S9), with an overall mean RMSE of 3.12 (Table S2).


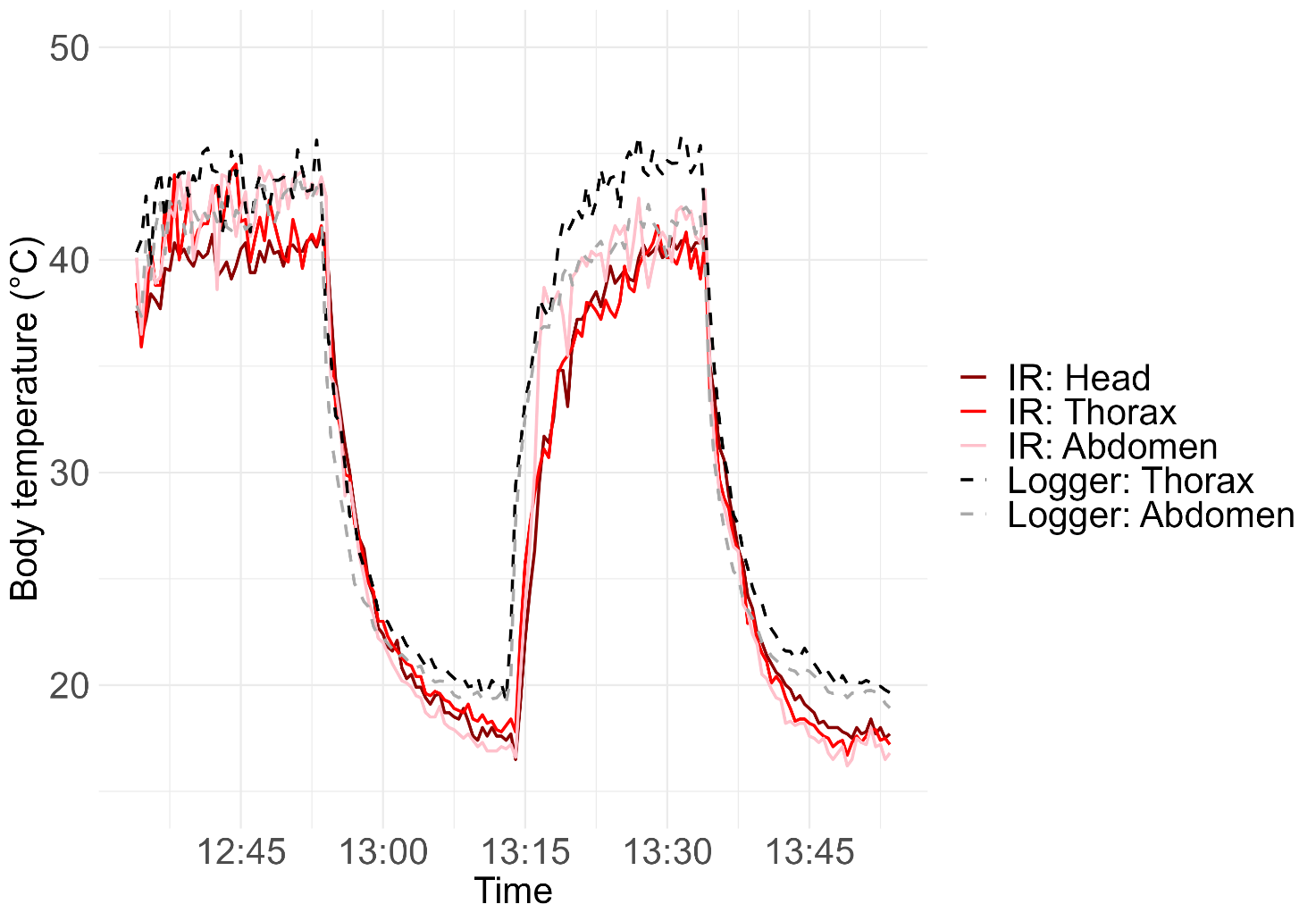


**Figure S9**: **Thermocouple (Logger: Thorax and Abdomen) and thermal camera (IR: Head, Thorax, Abdomen) body temperature measurements.**

**Table S2: RMSE values for the thermocouple (Logger thorax and abdomen) and thermal camera (IR head, thorax, and abdomen) measurements**

|  | **IR head** | **IR thorax** | **IR abdomen** |
| --- | --- | --- | --- |
| **Logger thorax** | 3.90 | 3.59 | 3.23 |
| **Logger abdomen** | 2.97 | 2.59 | 2.45 |
| **Mean RMSE** | **3.44** | **3.09** | **2.84** |

**References**

1. Anderson, R. V., Tracy, C. R., & Abramsky, Z. (1979). Habitat selection in two species of short-horned grasshoppers. *Oecologia, 38*(3), 359-374.
2. Buckley, L. B., Miller, E. F., & Kingsolver, J. G. (2013). Ectotherm thermal stress and specialization across altitude and latitude. *Integrative and comparative biology, 53*(4), 571-581.
3. Galushko, D., Ermakov, N., Karpovski, M., Palevski, A., Ishay, J. S., & Bergman, D. J. (2005). Electrical, thermoelectric and thermophysical properties of hornet cuticle. *Semiconductor science and technology, 20*(3), 286.
4. Gates, D.M. (1980). *Biophysical Ecology*. Courier Corporation.
5. Kearney, M. R., & Porter, W. P. (2017). NicheMapR–an R package for biophysical modelling: the microclimate model. *Ecography, 40*(5), 664-674.
6. Kearney, M. R., & Porter, W. P. (2020). NicheMapR–an R package for biophysical modelling: the ectotherm and Dynamic Energy Budget models. *Ecography, 43*(1), 85-96.
7. Piñeiro, G., Perelman, S., Guerschman, J. P., & Paruelo, J. M. (2008). How to evaluate models: observed vs. predicted or predicted vs. observed?. *Ecological modelling, 216*(3-4), 316-322.
8. Porter, W. P., & Gates, D. M. (1969). Thermodynamic equilibria of animals with environment. *Ecological monographs, 39*(3), 227-244.
9. R Core Team (2023). R: A language and environment for statistical computing. R Foundation for Statistical Computing.
10. Remmert, H. (1985). Crickets in sunshine. *Oecologia*, 29-33.
11. Wang, S., Tang, J., Cavalieri, R. P., & Davis, D. C. (2003). Differential heating of insects in dried nuts and fruits associated with radio frequency and microwave treatments. *Transactions of the ASAE, 46*(4), 1175.
